# Diagnostic accuracy of artificial intelligence for tuberculosis detection from cough sounds: a systematic review and meta-analysis

**DOI:** 10.3389/frai.2026.1774125

**Published:** 2026-06-23

**Authors:** Rakesh Kumar Sahoo, Krushna Chandra Sahoo, Abhinav Sinha, Rounik Talukdar, Milinda Mishra, Debdutta Bhattacharya, Sanghamitra Pati

**Affiliations:** 1 Health Technology Assessment India Regional Resource Hub, ICMR-Regional Medical Research Centre, Bhubaneswar, Odisha, India; 2 Health Technology Assessment India, Department of Health Research, Ministry of Health & Family Welfare, Govt. of India, New Delhi, India; 3 South Asian Institute of Health Promotion, Bhubaneswar, India; 4 National Cancer Institute, All India Institute of Medical Sciences, New Delhi, India

**Keywords:** artificial intelligence, cough sounds, diagnostic accuracy, systematic review, tuberculosis detection

## Abstract

Tuberculosis (TB) remains the leading cause of death from infectious diseases globally, with significant diagnostic challenges in low- and middle-income countries. Artificial intelligence (AI) analysis of cough sounds could offer an inexpensive and accessible solution for detecting TB. This systematic review and meta-analysis evaluated the diagnostic accuracy of AI-based cough sound analysis for screening TB and identified key methodological gaps. We performed a systematic review (PROSPERO: CRD420250656065), searching PubMed, Scopus, IEEE, Web of Science, and CINAHL for studies published between 1 January 2009 and 31 December 2024. Included studies focused on the application of AI-algorithms for TB screening based on cough sound analysis. Risk of bias was assessed using the QUADAS-AI tool. The sensitivity, specificity, and area under the curve were extracted to quantify diagnostic performance. Overall, 14 studies were found, largely from Asia and Africa. Although a meta-analysis of seven studies showed a pooled sensitivity of 91% (95% CI: 88–94%) and a specificity of 89% (95% CI: 85–92%), with a diagnostic odds ratio of 81.61 and an area under the curve of 0.9539, indicating strong diagnostic accuracy, most of the included studies focused on analytical validity. Artificial intelligence models for cough sounds might improve TB detection, particularly in resource-limited settings, by offering a non-invasive, rapid screening tool. However, the high risk of bias, heterogeneity, and reliance on internal validation highlights the need for multicenter clinical validity studies before adoption.

## 1 Introduction

Tuberculosis (TB) remains the foremost infectious disease-related cause of mortality globally; an estimated 10.7 million people developed TB, and 1.23 million died, with the burden concentrated in low- and middle-income countries (LMICs) (Nathavitharana et al., 2017; Chen et al., 2025; World Health Organization, 2025). Eight countries accounted for about two-thirds of incident cases (India, Indonesia, the Philippines, China, Pakistan, Nigeria, the Democratic Republic of the Congo, and Bangladesh), and the top five countries accounted for 55% of the global total (India, Indonesia, the Philippines, China, and Pakistan) (Nathavitharana et al., 2017; Chen et al., 2025; World Health Organization, 2025). Despite decades of control efforts, underfunding and diagnostic gaps persist, delaying treatment and sustaining transmission (The Global Fund, 2024; Lung et al., 2019). Sustainable Development Goal 3 (SDG 3) targets the elimination of TB by 2030 through prevention, improved treatment, and enhanced diagnostics (Merk et al., 2019). Achieving this goal requires innovation focusing on “emerging applications of artificial intelligence (AI) for predictive modelling in precision medicine” (Umar et al., 2024; Zhan et al., 2022; World Health Organization, 2014).

Effective TB control relies on accurate, timely diagnosis. Current approaches include rapid molecular assays (e.g., GeneXpert MTB/RIF), radiographic imaging (chest X-rays), and symptom-based screening (World Health Organization, 2015). While the *Mycobacterium tuberculosis* culture test remains the gold standard, its slow turnaround and infrastructure needs limit its use in resource-poor settings. The GeneXpert MTB/RIF assay allows rapid identification of TB DNA (tuberculosis deoxyribonucleic acid) and drug resistance; however, in 2022, it was used as the initial test in only 33% of patients (Zhan et al., 2022). Although rapid molecular diagnostics are effective, their widespread adoption is constrained by high costs, equipment demands, and limited accessibility (Umar et al., 2024; Zhan et al., 2022; World Health Organization, 2014). The World Health Organization (WHO) recommends chest X-rays as low-cost, sensitive screening tools; however, the accuracy depends on radiologist expertise, and access is limited in many primary care facilities (World Health Organization, 2014; World Health Organization, 2015), which brings a challenge in TB detection and early treatment. Furthermore, symptom-based screening, such as cough questionnaires, has only 42% sensitivity and faces challenges in low-resource settings. In contrast, WHO recommends TB triage tests for communities to meet at least 90% sensitivity and 70% specificity (World Health Organization, 2014), prompting the exploration of novel, accessible tools.

The WHO End-TB strategy emphasized innovative methods like digital cough monitoring and acoustic epidemiology, enhancing community-based TB screening (World Health Organization, 2015; Houben et al., 2016; Zimmer et al., 2022). In pulmonary TB, cough, a key symptom, differs acoustically from that of healthy individuals and other respiratory diseases due to distinct glottic biomechanics (Chung and Pavord, 2008; Simonsson et al., 1967; Turner and Bothamley, 2015; Kaplan, 2019; Botha et al., 2018; Pahar et al., 2021). Leveraging sound analysis and acoustic pattern recognition, AI offers a highly efficient diagnostic pathway. Ubiquitous computing, including deep learning (DL) and machine learning (ML), has been applied in COVID-19 and other respiratory disease prediction through sound analysis (Proano et al., 2016; Topalovic et al., 2019; Lella and Pja, 2021; Bao et al., 2023). AI-driven cough analysis is revolutionizing TB detection, enabling early intervention and reduced laboratory dependence. Previous studies have examined AI-based methods for TB diagnosis, like radiographic imaging, AI-powered tools, and broader TB-biomarkers (Hansun et al., 2025). However, there is limited systematic evidence on the diagnostic performance of AI algorithms based on the cough sound analysis. This review evaluated the diagnostic accuracy of AI-based cough sound analysis for screening TB.

## 2 Methods

### 2.1 Protocol and registration

The present systematic review was appropriately registered in the International Prospective Register of Systematic Reviews (PROSPERO; registration ID: CRD420250656065). Established reporting frameworks, such as the PRISMA guidelines, were employed to conduct the review (Page et al., 2021). Outlining of detailed methods for the sensitivity and subgroup analyses is provided (Supplementary Figure S1).

### 2.2 Databases and search strategy

A structured literature search across the electronic databases Scopus, PubMed, IEEE Xplore, Web of Science (WOS), and Cumulative Index to Nursing and Allied Health Literature (CINAHL) was conducted. The search was restricted to publications in English from January 1, 2009, to December 31, 2024. The search strategy was developed around key terms and concepts: “Artificial Intelligence (AI),” “Machine Learning (ML),” “Deep Learning (DL),” “Tuberculosis,” “Pulmonary Tuberculosis,” “TB,” “Cough Sound Analysis,” and “Audio Cough.” The search strategy was customized for each database, with detailed descriptions provided (Supplementary Table S1). The study selection was conducted independently by two reviewers, who adhered to predefined eligibility criteria.

### 2.3 Eligibility criteria

Studies were deemed eligible if they reported quantitative measures of diagnostic performance, including sensitivity, specificity, and overall classification accuracy, or if they presented a complete 2 × 2 confusion matrix, detailing true positive (TP), true negative (TN), false positive (FP), and false negative (FN) outcomes. Studies were excluded if they were identified as duplicates, employed irrelevant research methodologies (including individual case narratives, experimental design studies, review articles, or conference abstracts), provided insufficient data, or failed to report the crucial diagnostic outcomes. Table 1 shows the population, intervention, comparison, outcomes and study design (PICOS).

**Table 1:** Population, intervention, comparison, outcomes, and study design.

Population (P)	Studies involving individuals with confirmed or suspected tuberculosis and healthy participants.
Intervention (I)	Use of AI-based algorithms (ML or DL) for cough sound analysis to detect TB.
Comparison (C)	Reference standard diagnostic methods such as GeneXpert MTB/RIF, Acid-Fast Bacilli (AFB) smear microscopy, culture, Cartridge-Based Nucleic Acid Amplification Test (CB-NAAT), chest X-rays, or CT scans.
Outcomes (O)	Diagnostic performance measures, including sensitivity, specificity, and overall classification accuracy, or sufficient data to construct a 2 × 2 contingency table (TP, FP, TN, FN).
Study design (SD)	*Eligible designs:* Observational studies, experimental studies, and diagnostic accuracy studies reporting quantitative performance metrics. *Excluded designs:* Case reports, narrative reviews, systematic reviews, conference abstracts, studies without relevant diagnostic outcomes, and those with insufficient or missing performance data.

### 2.4 Data extraction and quality assessment

A structured and predefined data extraction sheet, including sound acquisition and cough event detection, eligibility criteria, feature engineering and modelling, study context, diagnostic accuracy results, and area under the curve, was used. Discrepancies identified during the data extraction process were resolved by mutual agreement within the research team. In instances of incomplete or missing information, we contacted the corresponding author to obtain the necessary clarification. The Quality Assessment of Diagnostic Accuracy Studies—Artificial Intelligence instrument was used to evaluate the quality of the included studies (Moons et al., 2014; Sounderajah et al., 2021). The analytical validation model is evaluated offline/retrospectively under controlled conditions, often using an existing dataset. It checks technical performance (e.g., AUC, sensitivity/specificity) without testing real-world clinical workflow/use. The data may still come from patients, but it’s not validated in routine care conditions. However, in the case of clinical validation, the model is tested on real patients in the intended clinical setting/workflow, compared against a clinical reference standard, to show it performs reliably in clinical practice.

### 2.5 Statistical analysis

The diagnostic performance of the AI architectures was evaluated through a hierarchical summary receiver operating characteristic (SROC) analysis, yielding pooled estimates with corresponding 95% confidence intervals for key metrics, including sensitivity, specificity, Diagnostic Odds Ratio (DOR), and Area Under the Curve (AUC). In instances where multiple algorithms were assessed within a single study, we extracted a complete 2 × 2 contingency table (TP, FP, FN, TN) for each algorithm and included all models with complete 2 × 2 data in the meta-analysis; models without sufficient 2 × 2 information were excluded. The degree of heterogeneity among the included studies was assessed using the I-square statistic. Subgroup analyses were conducted to investigate underlying sources of heterogeneity across the included studies. In light of the expected inter-study variation, a random-effects modelling approach was adopted for the meta-analytic synthesis. Potential publication bias was evaluated through visual inspection of funnel plot asymmetry and corroborated using regression-based statistical tests.

To strengthen the robustness of the findings, positive and negative likelihood ratios (LR positive and LR negative) were calculated, offering critical information on the diagnostic utility of the tool for both confirming and ruling out disease across diverse clinical settings, thus reinforcing its relevance for informed clinical decision-making. Subgroup analyses were stratified into three categories: type of AI algorithm applied such as machine learning or deep learning; performance based on sample size (≤1,000 or >1,000); and geographic distribution (Asia or non-Asia regions). For the geographic subgroup analysis, only studies conducted entirely within one geographic category were included; the multicentric study involving both Asian and non-Asian countries was excluded from this subgroup comparison but retained in the overall meta-analysis. Additionally, pooled sensitivity and specificity were jointly estimated using a bivariate random-effects meta-analysis (Reitsma model). Maximum likelihood estimation was used to estimate the model’s parameters. Statistical synthesis was carried out using MetaDisc (version 1.4, Spain) and Stata (version 17, United States), applying a two-tailed significance level of *α* = 0.05. To visually depict the distribution of sensitivity and specificity across the included studies, a crosshair plot was generated using Python (v3.8.18, Netherlands) (Phillips et al., 2010).

## 3 Results

### 3.1 Study selection and characteristics

**Figure 1:**
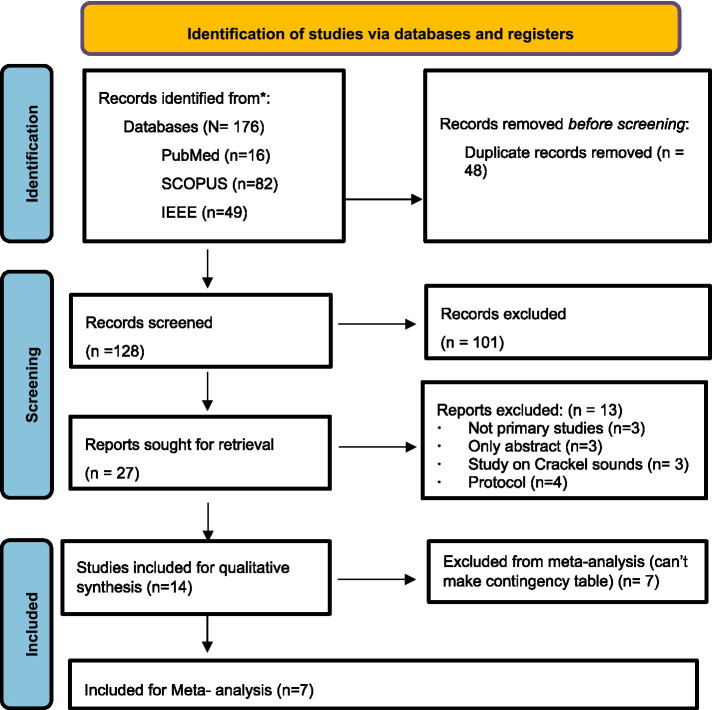
PRISMA flow diagram.

A total of 176 articles were retrieved from five databases. After the removal of 48 duplicate records, 128 studies proceeded to the screening phase. Title and abstract screening led to the exclusion of 101 records based on relevance and eligibility criteria. Subsequently, 27 full-text articles were assessed for inclusion. Following a detailed full-text assessment, 14 studies (Pahar et al., 2021; Bao et al., 2023; Frost et al., 2022; Huddart et al., 2024; Jember et al., 2023; Malik and Anees, 2024; Malik et al., 2023; Pahar et al., 2022a; Pahar et al., 2022b; Tracey et al., 2011; Xu et al., 2024a; Xu et al., 2024b; Yellapu et al., 2023; Yuan et al., 2023) were included in the qualitative synthesis, of which seven studies (Bao et al., 2023; Frost et al., 2022; Huddart et al., 2024; Jember et al., 2023; Tracey et al., 2011; Xu et al., 2024a; Yellapu et al., 2023) were included in the meta-analysis (Figure 1).

**Figure 2:**
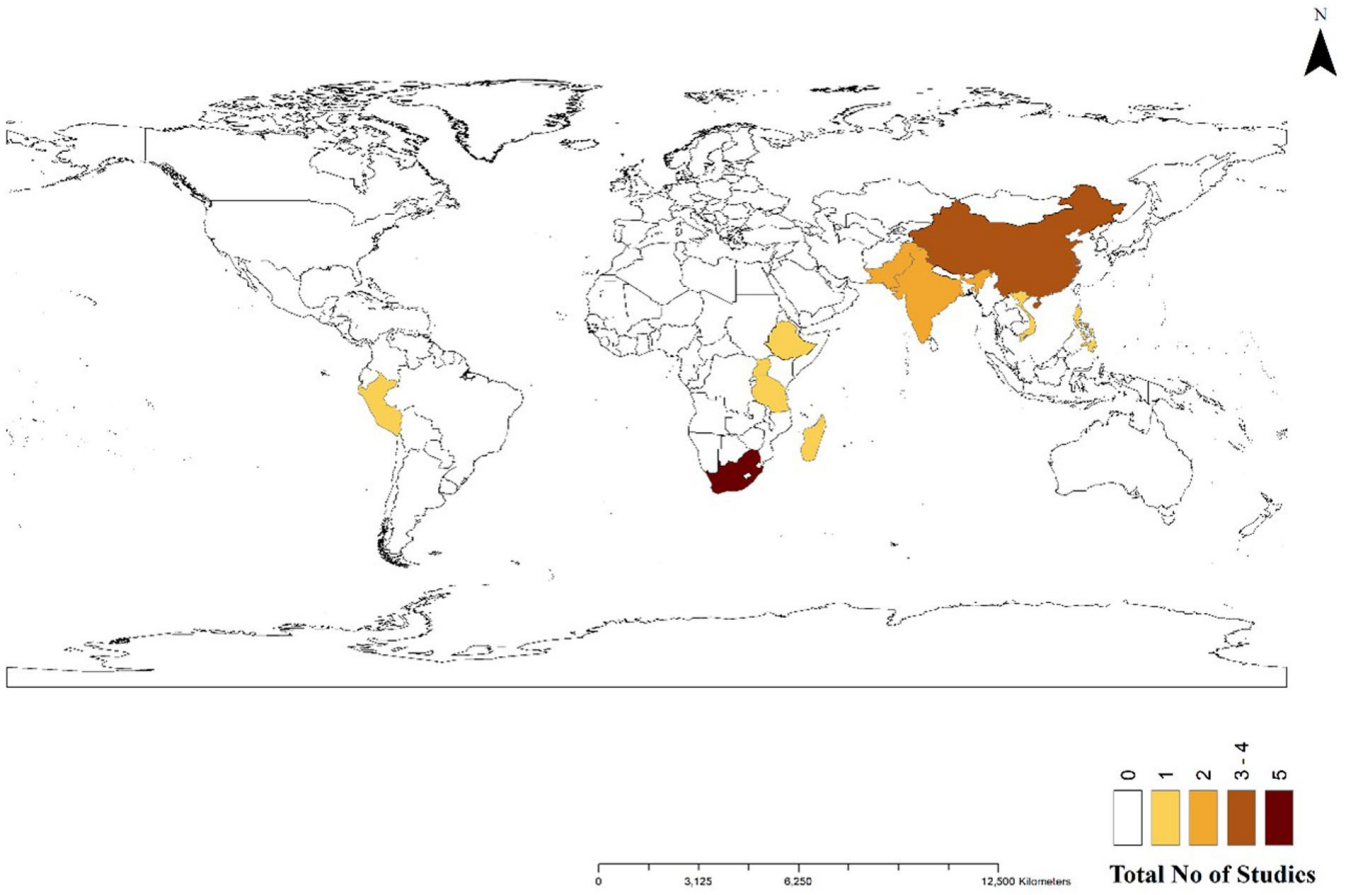
Distribution of the studies across the globe.

The geographical distribution of the included studies (Figure 2) spanned Asia, Africa, and South America: four were conducted in China (Bao et al., 2023; Xu et al., 2024a; Xu et al., 2024b; Yuan et al., 2023), one in India (Yellapu et al., 2023), and two in Pakistan (Malik and Anees, 2024; Malik et al., 2023); Africa contributed four studies from South Africa (Pahar et al., 2021; Frost et al., 2022; Pahar et al., 2022a; Pahar et al., 2022b) and one from Ethiopia (Jember et al., 2023); one study was from Peru (Tracey et al., 2011). In addition, one large multi-country study included participants from India, Madagascar, the Philippines, South Africa, Tanzania, Uganda, and Vietnam (Huddart et al., 2024).

Out of the 14 studies, ten collected data prospectively from various hospital-based outpatient clinics (Pahar et al., 2021; Bao et al., 2023; Frost et al., 2022; Huddart et al., 2024; Pahar et al., 2022a; Pahar et al., 2022b; Tracey et al., 2011; Xu et al., 2024a; Xu et al., 2024b; Yellapu et al., 2023), ensuring real-time data acquisition (Table 2). Three studies adopted a retrospective approach, analyzing previously recorded datasets from online databases and site-based recording (Frost et al., 2022; Malik and Anees, 2024; Malik et al., 2023). In contrast, one study utilized a combined retrospective and prospective data collection method to enhance dataset comprehensiveness (Pahar et al., 2022a). In ten studies, prospective data collection was undertaken across multiple clinical environments, including outpatient clinics, inpatient wards, and primary healthcare settings. None of the studies used a soundproof room during data collection. However, ten studies maintained relative silence or a quiet clinical environment to minimize background noise during recording, two studies conducted recordings in a noise-isolated facility, ensuring controlled acoustic conditions (Frost et al., 2022; Huddart et al., 2024), and one study collected longitudinal cough data in real-world community settings, capturing cough sounds in uncontrolled, natural environments.

**Table 2:** Study characteristics.

Study	Country	Participants (*N*)	The device captures the sound	Settings and data type	Soundproof room for sample collection	TB patients confirmation	Number of cough events	Feature extraction technique	Algorithms	AI Algorithm architecture	Accuracy measure	Types of validation
Tracey et al. (2011)	Peru	TB patients with drug-susceptible, MDR and HIV coinfection (*N* = 62)	Marantz PMD 620 handheld recorder with Audio-Technica AT899 sub-mini microphone	Hospital-based Prospective Study	No	Sputum smear microscopy, MODS culture	418	MFCCs	ML	neural networks (MLP), SVM, SMO	TP, TN, FP, FN, Sn, Sp, AUC, ACC	Analytical
Huddart et al. (2024a)	India, Madagascar, Philippines, South Africa, Tanzania, Uganda, and Vietnam	Presumptive tuberculosis outpatient (*N* = 2,143)	Hyfe Research App (smartphones)	Hospital and Community-based Prospective Study	Yes (solicited cough collection), No (longitudinal cough collection)	Microbiologic reference standard, Xpert MTB/RIF Ultra standard	7,33,756	Log Mel Spectrogram images	DL	VGG16	Sn, Sp, TP, TN, FP, FN	Analytical
Pahar et al. (2022a)	South Africa	Patients of TB, COVID-19, Healthy Individuals (*N* = 1774)	Smartphones equipped with external microphones (RØDE M3, RØDE M1)	Hospital and Community-based Prospective & Retrospective Study	No	Standard clinical diagnostic protocols	NA	MFCCs	DL	ResNet50-TL, CNN-TL, LSTM-TL	F1-Score, AUC, Sn, Sp, ACC	Analytical
Yuan et al. (2023)	China	TB, other respiratory diseases, and healthy individuals (*N* = 345)	Smartphones	Hospital-based Prospective Study	No	IGRA, PPD, Recombinant *Mycobacterium tuberculosis* fusion protein	1,323	MFCCs, Mel Spectrograms	DL	CNN + Feedforward Neural Network.	F1-Score, AUC, Sn, Precision	Analytical
Pahar et al. (2022b)	South Africa	TB and non-TB patients (*N* = 48)	ZOOM F8N field recorder with RØDE M3 condenser microphone	Hospital-based Prospective Study	No	Standardized TB diagnostic methods	1,235	MFCCs, ZCR, kurtosis, NLP-style cough embeddings	DL	LSTM, CNN, SVM, MLP, LR, KNN	F1-Score, AUC	Analytical
Frost et al. (2022)	South Africa	TB-positive and TB-negative patients (*N* = 74)	NA	Hospital-based retrospective study	Both: (Brooklyn dataset collected in a noise-isolated facility, Wallacedene dataset in a noisy environment)	Sputum analysis	1,564	Mel-spectrograms, linear filter-bank energies, MFCCs, neural style transfer	ML & DL	LR (baseline), BiLSTM, BiLSTM (SFS), BiLSTM-Att, BiLSTM-Att (SFS)	Sn, Sp, AUC, ACC, TP, TN, FP, FN	Analytical
Bao et al. (2023)	China	TB-positive and TB-negative participants (*N* = 356)	Smartphone	Hospital-based Prospective Study	NA	standard clinical test	1,000	MFCCs, ZCR, Short-Time Energy, RMS, chromocene	DL	Resnet50, Googlenet, Bi-LSTM and Google net	ACC, Sn, Sp, TP, TN, FP, FN	Analytical
Jember et al. (2023)	Ethiopia	Patients with various respiratory diseases (*N* = 71)	Philips DVT1200 Digital Voice Recorder, HM1000 Microphone and Infinix Hot 8 Smartphone (Model X650C)	Hospital-based Prospective Study	No	GeneXpert, X-ray, CT, Bronchoscopy	6,476	MFCCs	DL	ANN, SVM	TP, TN, FP, FN, ACC, F1-Score	Analytical
Xu et al. (2024a)	China	Patients with tuberculosis and healthy individuals (*N* = 144)	Smartphone	Hospital-based Prospective Study	No	Xpert MTB/RIF, IGRA, PPD	456	MFCC, ZCR, Short-Time Energy, RMS, Chroma_cens	DL	Bi-LSTM, Bi-GRU, LSTM, GRU, Conv2D, Bi-LSTM+Conv2d	TP, TN, FP, FN, ACC, Sn, Sp, AUC	Analytical
Xu et al. (2024b)	China	Patients with TB, other respiratory diseases, and healthy individuals (*N* = 345)	Smartphone	Hospital-based Prospective Study	No	X-ray, IGRA, PPD, Recombinant *Mycobacterium tuberculosis* Fusion Protein	1,323	Mel spectrograms, Spectrogram Conversion	DL	DMRNet, DMRNet, ResNet34, ResNet50, DenseNet121, Google Net, InceptionV3, VGG, Xception, Mobile Net	ACC, F1-Score, Sn, Sp	Analytical
Pahar et al. (2021)	South Africa	Patients with respiratory diseases (*N* = 51)	ZOOM F8N Recorder and RØDE M3 Microphone	Hospital-based Prospective Study	No	Bacteriological TB diagnosis	1,358	MFCCs, Log-Filterbank Energies, ZCR, Kurtosis	ML & DL	LR, KNN, SVM, MLP, CNN	AUC, Sn, Sp, ACC	Analytical
Yellapu et al. (2023)	India	pulmonary TB patients (*N* = 567)	Smartphones & Tablets	Hospital-based Prospective Study	No	CB-NAAT, X-ray, Acid-Fast Bacilli Staining	3,102	MFCCs, ZCR, Spectral Features, Chroma	DL	Hybrid CNN + FFANN	TP, TN, FP, FN, Sn, Sp, AUC, PPV, NPV	Both Analytical and Clinical
Malik et al. (2023)	Pakistan	chest diseases such as COVID-19, TB, pneumonia, lung cancer (*N* = NA)	Smartphones	Retrospective Records	No	NA	1,194	Scalogram (CWT), MFCCs, noise filtering	DL	DenseNet-201, EfficientNet-B0, InceptionResNet-V2, Xception, Proposed Model (with BL-SMOTE)	ACC, F1-Score, Precision, Sn, AUC	Analytical
Malik and Anees (2024)	Pakistan	chest disease participants (*N* = 51)	Mobile devices with external microphones	Retrospective Records	No	X-ray and CT	402	Scalogram transformation, deep convolutional feature extraction	DL	VGG-19, ResNet-101, ResNet-50, DenseNet-121, Inception-V3, EfficientNetB0, DenseNet-201, Mode lP (4)	ACC	Analytical

TB: Tuberculosis; MDR: multidrug-resistant; HIV: human immunodeficiency virus; IGRA: interferon-gamma release assay; PPD: purified protein derivative (tuberculin skin test reagent); MODS: Microscopic Observation Drug Susceptibility (assay/culture); Xpert MTB/RIF Ultra: Xpert test for *Mycobacterium tuberculosis* (MTB) detection and rifampicin (RIF) resistance (Ultra cartridge); CB-NAAT/CBNAAT: cartridge-based nucleic acid amplification test; CT: computed tomography; NA: not available/not reported; MFCCs: mel-frequency cepstral coefficients; ZCR: zero-crossing rate; STFT: short-time Fourier transform; CWT: continuous wavelet transform; RMS: root mean square; STE: short-time energy; ML: machine learning; DL: deep learning; NLP: natural language processing; ANN: artificial neural network; MLP: multilayer perceptron; SVM: support vector machine; SMO: sequential minimal optimization; LR: logistic regression; KNN/kNN: k-nearest neighbors; CNN: convolutional neural network; LSTM: long short-term memory; GRU: gated recurrent unit; BiLSTM/Bi-LSTM: bidirectional LSTM; Bi-GRU: bidirectional GRU; Conv2D: 2-dimensional convolution layer; TL: transfer learning; FFANN: feed-forward artificial neural network; VGG (VGG16/VGG-19): Visual Geometry Group network; ResNet (ResNet34/50/101): residual network; DenseNet (DenseNet121/201): densely connected convolutional network; InceptionV3: Inception version 3; InceptionResNet-V2: Inception-ResNet version 2; Xception: extreme inception architecture; MobileNet: mobile network architecture; EfficientNet-B0: EfficientNet baseline (B0); DMRNet: author-named model (not expanded in the source paper); BL-SMOTE/BLSMOTE: borderline synthetic minority over-sampling technique; SFS: sequential forward selection; TP: true positive; TN: true negative; FP: false positive; FN: false negative; Sn: sensitivity; Sp: specificity; AUC: area under the (ROC) curve; ACC: accuracy; F1-score: harmonic mean of precision and recall; PPV: positive predictive value; NPV: negative predictive value.

Cough sounds were recorded from TB-confirmed patients, with diagnosis established through sputum smear microscopy (Frost et al., 2022; Tracey et al., 2011), GeneXpert MTB/RIF (Huddart et al., 2024; Jember et al., 2023; Xu et al., 2024a), acid-fast bacilli (AFB) staining (Yellapu et al., 2023), bacteriological TB diagnosis, chest X-rays (CXR) (Pahar et al., 2021; Malik and Anees, 2024), and computed tomography (CT) scans (Jember et al., 2023; Malik and Anees, 2024). In certain circumstances, cartridge-based nucleic acid amplification tests (CB-NAAT) (Yellapu et al., 2023) and recombinant *Mycobacterium tuberculosis* fusion protein assays (Xu et al., 2024b; Yuan et al., 2023) were used to improve diagnostic accuracy. To ensure model robustness and reduce classification bias, cough recordings were also collected from non-TB individuals, enabling better differentiation between TB-positive and TB-negative cases. All studies used smartphones, smartphones with external microphones (RØDE M3, ZOOM F8N, HM 1000) (Pahar et al., 2021; Jember et al., 2023; Pahar et al., 2022a; Pahar et al., 2022b), and dedicated recording devices with microphones (Marantz PMD 620, Audio-Technica AT899) for cough sound capture (Tracey et al., 2011). Recordings were conducted in outpatient clinics and community settings, with microphones positioned strategically for optimal capture.

Raw cough sounds contain complex acoustic signals, making feature extraction essential for enhancing data interpretability in AI models. Mel Frequency Cepstral Coefficients (MFCCs) were the most commonly used feature extraction technique (*n* = 3) because of their ability to capture spectrum features (Jember et al., 2023; Pahar et al., 2022a; Tracey et al., 2011). Additionally, Log Mel Spectrograms were used in two studies (*n* = 2) for feature extraction (Pahar et al., 2021; Huddart et al., 2024). Several other studies adopted hybrid feature extraction approaches, combining MFCCs with Zero-Crossing Rate (ZCR), Short-Time Fourier Transform (STFT), Root Mean Square (RMS), Short-Time Energy (STE), Scalograms, Chroma Features, and Filter-Bank Energies to enhance AI-driven disease detection (Table 2).

Out of the fourteen included studies, eleven exclusively employed deep learning (DL) methods (Bao et al., 2023; Huddart et al., 2024; Jember et al., 2023; Malik and Anees, 2024; Malik et al., 2023; Pahar et al., 2022a; Pahar et al., 2022b; Xu et al., 2024a; Xu et al., 2024b; Yellapu et al., 2023; Yuan et al., 2023), one used only ML methods (Tracey et al., 2011), and two incorporated a combination of both ML and DL approaches (Pahar et al., 2021; Frost et al., 2022). In contrast, others employed hybrid architectures based solely on deep learning methods for cough sound classification (Frost et al., 2022; Malik et al., 2023; Xu et al., 2024a; Xu et al., 2024b; Yellapu et al., 2023). The detailed AI algorithm architecture of each study is presented in Table 2.

### 3.2 Quality assessment

**Figure 3:**
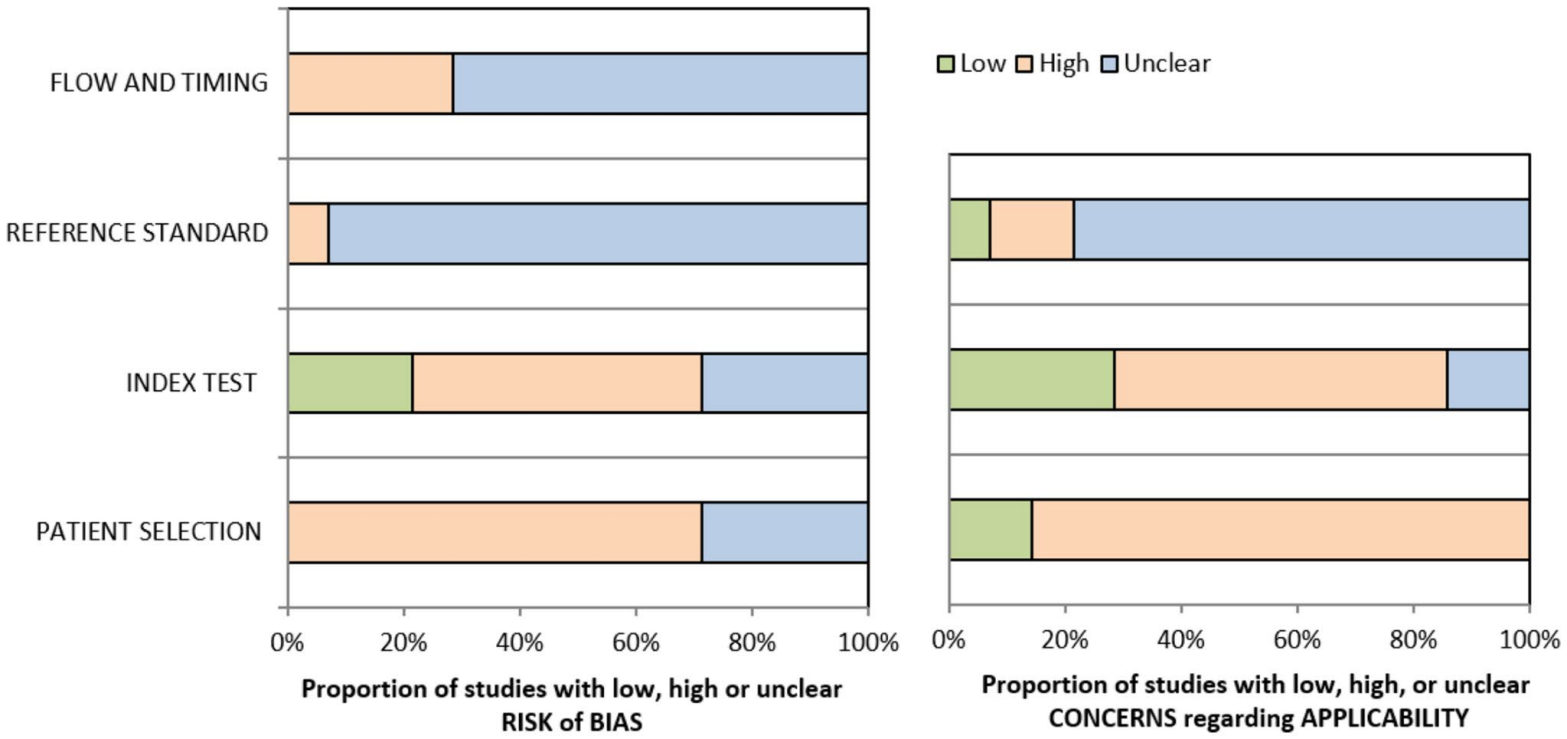
QUADAS-AI summary plot for risk-bias assessment.

Overall, the quality assessment indicated a substantial risk of bias and notable applicability concerns across the 14 studies that were included (Pahar et al., 2021; Bao et al., 2023; Frost et al., 2022; Huddart et al., 2024; Jember et al., 2023; Malik and Anees, 2024; Malik et al., 2023; Pahar et al., 2022a; Pahar et al., 2022b; Tracey et al., 2011; Xu et al., 2024a; Xu et al., 2024b; Yellapu et al., 2023; Yuan et al., 2023). Risk-of-bias appraisal showed that patient selection was commonly rated high risk in ten studies (Bao et al., 2023; Huddart et al., 2024; Jember et al., 2023; Malik and Anees, 2024; Malik et al., 2023; Pahar et al., 2022a; Xu et al., 2024a; Xu et al., 2024b; Yellapu et al., 2023; Yuan et al., 2023), with the remaining studies judged unclear (Pahar et al., 2021; Frost et al., 2022; Pahar et al., 2022b; Tracey et al., 2011). For the index test, low risk was observed in three studies (Frost et al., 2022; Huddart et al., 2024; Yellapu et al., 2023), whereas assessments were unclear in four studies (Bao et al., 2023; Jember et al., 2023; Malik and Anees, 2024; Xu et al., 2024a) and high risk in the remaining studies (Pahar et al., 2021; Malik et al., 2023; Pahar et al., 2022a; Pahar et al., 2022b; Tracey et al., 2011; Xu et al., 2024b; Yuan et al., 2023). The reference standard domain was predominantly rated as having an unclear risk (Pahar et al., 2021; Bao et al., 2023; Frost et al., 2022; Huddart et al., 2024; Jember et al., 2023; Malik and Anees, 2024; Pahar et al., 2022a; Pahar et al., 2022b; Tracey et al., 2011; Xu et al., 2024a; Xu et al., 2024b; Yellapu et al., 2023; Yuan et al., 2023), while one study (Malik et al., 2023) was identified as having a high risk. Flow and timing raised particular concerns in Huddart et al. (2024), Malik and Anees (2024), Malik et al. (2023), and Tracey et al. (2011), which were judged high risk, while the remaining studies were assessed as unclear (Pahar et al., 2021; Bao et al., 2023; Frost et al., 2022; Jember et al., 2023; Pahar et al., 2022a; Pahar et al., 2022b; Xu et al., 2024a; Xu et al., 2024b; Yellapu et al., 2023; Yuan et al., 2023). Applicability concerns were also notable: patient selection was largely judged high concern (Bao et al., 2023; Frost et al., 2022; Huddart et al., 2024; Jember et al., 2023; Malik and Anees, 2024; Malik et al., 2023; Pahar et al., 2022a; Tracey et al., 2011; Xu et al., 2024a; Xu et al., 2024b; Yellapu et al., 2023; Yuan et al., 2023), with low concern in Pahar et al. (2021) and Pahar et al. (2022b); index test applicability was low in Bao et al. (2023), Jember et al. (2023), Xu et al. (2024a), and Yellapu et al. (2023), unclear in Frost et al. (2022) and Huddart et al. (2024), and high in Pahar et al. (2021); Malik and Anees (2024), Malik et al. (2023), Pahar et al. (2022a), Pahar et al. (2022b), Tracey et al. (2011), Xu et al. (2024b), and Yuan et al. (2023); and reference standard applicability was mostly unclear (Pahar et al., 2021; Bao et al., 2023; Frost et al., 2022; Jember et al., 2023; Pahar et al., 2022a; Pahar et al., 2022b; Tracey et al., 2011; Xu et al., 2024a; Xu et al., 2024b; Yellapu et al., 2023; Yuan et al., 2023), with high concerns in Malik and Anees (2024) and Malik et al. (2023) and low concern in Huddart et al. (2024) (Supplementary Table S2, Figure 3).

### 3.3 Pooled accuracy of AI-analysed cough sound

**Figure 4:**
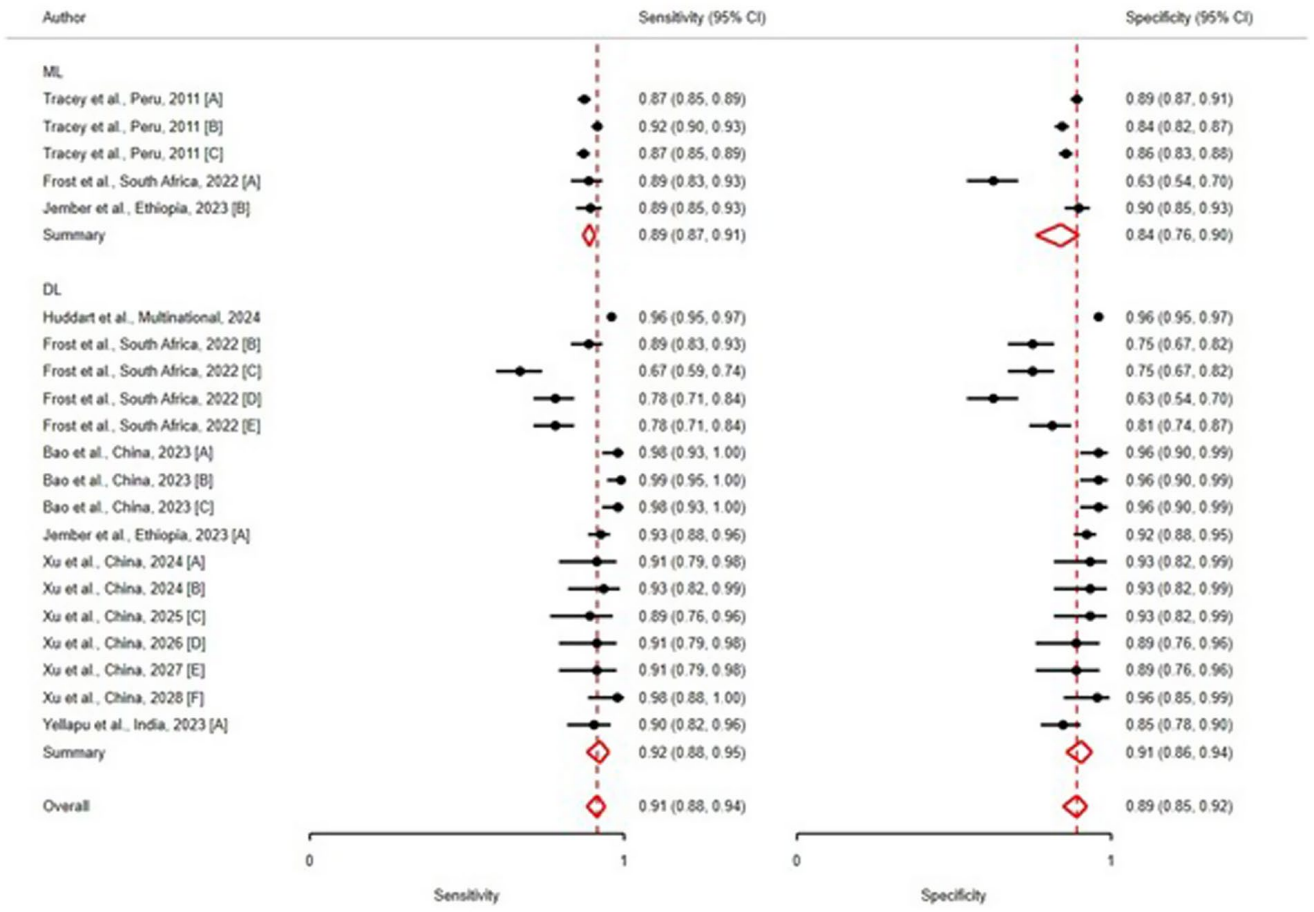
Forest plot of both deep learning (DL) and machine learning (ML).

Across the seven studies (Bao et al., 2023; Frost et al., 2022; Huddart et al., 2024; Jember et al., 2023; Tracey et al., 2011; Xu et al., 2024a; Yellapu et al., 2023) included in the meta-analysis (containing 21 distinct contingency tables), we found AI-analysed cough sounds to have a sensitivity of 91% (95% CI: 88–94%) and a specificity of 89% (95% CI: 85–92%) (Figure 4). The diagnostic odds ratio (DOR), indicating overall diagnostic accuracy, was calculated as 81.61 (95% CI: 41.01–162.37), and the summary receiver operating characteristic (SROC) curve produced an area under the curve (AUC) of 0.9539 (Supplementary Figure S2) and a crosshair plot of overall AI model performance (Supplementary Figure S3). Concerning the accuracy of different AI cough-sound interpretation models, deep learning models were found to be 92% (95% CI: 0.88–0.96) sensitive and 91% (95% CI: 0.86–0.94) specific (Bao et al., 2023; Huddart et al., 2024; Jember et al., 2023; Xu et al., 2024a; Yellapu et al., 2023). The diagnostic odds ratio (DOR) was 114.37 (95% CI: 36.32–360.19) and the AUC of 0.9557. In contrast, the sensitivity and specificity values of the machine learning models (Frost et al., 2022; Tracey et al., 2011) were 89% (95% CI: 0.87–0.91) and 84% (95% CI: 0.76–0.90), respectively. The corresponding DOR for these models was 43.32 (95% CI: 29.25–64.16), with an AUC of 0.945 (Supplementary Figure S4).

### 3.4 Subgroup analysis

Models trained on smaller datasets (*n* = 3, ≤1,000 samples) achieved pooled sensitivity of 90% (95% CI: 0.88 0.92) and specificity of 88% (95% CI: 0.86–0.90), alongside a high DOR of 116.89 (95% CI: 72.73–187.85) and an AUC of 0.9629 (Bao et al., 2023; Tracey et al., 2011; Xu et al., 2024a). Conversely, larger datasets (*n* = 4, >1,000 samples) yielded marginally higher sensitivity of 93% (95% CI: 0.89–0.98), and specificity of 92% (95% CI: 0.86–0.98), though the DOR was comparatively lower at 33.63 (95% CI: 7.89–143.31), with an AUC of 0.9311 (Frost et al., 2022; Huddart et al., 2024; Jember et al., 2023; Yellapu et al., 2023) (Supplementary Figure S5). In studies conducted in the Asia region (*n* = 3), sensitivity and specificity reached 95% (95% CI: 0.92–0.97) and 92% (95% CI: 0.89–0.95), respectively (Bao et al., 2023; Xu et al., 2024a; Yellapu et al., 2023). The diagnostic odds ratio (DOR) was significantly high at 236.30 (95% CI: 101.82–548.43), suggesting strong diagnostic capability with an AUC of 0.9704 (Bao et al., 2023; Xu et al., 2024a; Yellapu et al., 2023). In contrast, models evaluated in non-Asian settings (*n* = 3) exhibited lower sensitivity at 87% (95% CI: 0.84–0.91) and specificity at 84% (95% CI: 0.80–0.89), with a corresponding DOR of 27.51 (95% CI: 15.68–48.28) and an AUC of 0.9147 (Frost et al., 2022; Jember et al., 2023; Tracey et al., 2011) (Supplementary Figure S6). The multicentric study by Huddart et al. (2024), which included participants from both Asian and non-Asian countries, was retained in the overall pooled analysis but excluded from the region-based subgroup comparison to prevent erroneous geographic misclassification. A detailed comparison of pooled sensitivity, specificity, DOR, and likelihood ratios for AI-driven tuberculosis detection models based on cough sounds, stratified by algorithm category, sample size, and geographic region, is provided in Table 3.

**Table 3:** Summary of pooled diagnostic accuracy estimates for AI-based tuberculosis detection using cough sounds.

	No of studies	Sensitivity			Specificity			Diagnostic odds ratio			Likelihood ratio	
		Sensitivity	I2 value (%)	*p*-value	Specificity	I2 value (%)	*p*-value	DOR	I2 value (%)	*p*-value	LR+	LR-
Overall	7	0.91 (0.88–0.94)	94.4	0.0000	0.89 (0.85–0.92)	96.1	0.0000	81.61(41.01–162.37)	97.2	0.0000	8.01(5.37–11.97)	0.11(0.07–0.16)
Algorithm												
Machine learning (ML)	3	0.89 (0.87–0.91)	70.1	0.0097	0.84 (0.76–0.90)	93.6	0.0000	43.32 (29.25–64.16)	84.6	0.0000	5.62 (3.81–8.31)	0.13 (0.11–0.16)
Deep learning (DL)	4	0.92 (0.88–0.96)	94.5	0.0000	0.91 (0.86–0.94)	95.2	0.0000	114.37 (36.32–360.19)	97.5	0.0000	9.45 (4.93–18.08)	0.09 (0.04–0.17)
Sample size (> 1,000 & ≤ 1,000)												
≤ 1,000	3	0.90(0.88–0.92)	81.4	0.0000	0.88 (0.86–0.90)	77.5	0.0000	116.89 (72.73–187.85)	79.2	0.0000	9.40 (7.31–12.08)	0.10 (0.07–0.13)
> 1,000	4	0.93 (0.89–0.98)	96.9	0.0000	0.92(0.86–0.98)	98.1	0.0000	33.63 (7.89–143.31)	98.7	0.0000	5.20 (2.22–12.21)	0.16 (0.07–0.35)
Region classification												
Asia region	3	0.95 (0.92–0.97)	54.4	0.0195	0.92(0.89–0.95)	50.3	0.0338	236.30(101.82–548.43)	66.4	0.0015	13.17(8.38–20.70)	0.06 (0.04–0.10)
Non-Asia region	3	0.87 (0.84–0.91)	90.6	0.0000	0.84 (0.80–0.89)	93.5	0.0000	27.51 (15.68–48.28)	94.7	0.0000	4.69 (3.34–6.59)	0.17 (0.12–0.24)

*The multicentric study (Huddart et al., 2024) was included in the overall analysis but excluded from the region-based subgroup analysis because it included both Asian and non-Asian study sites.

**Figure 5:**
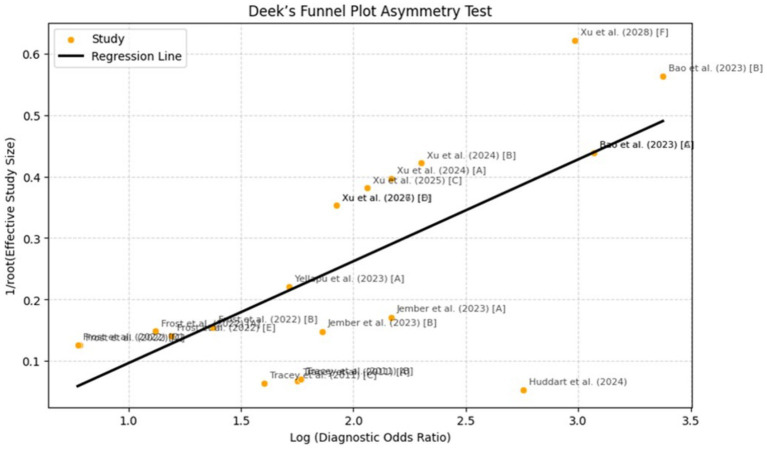
Publication bias plot.

Heterogeneity was high, with an I^2^ of 94.4% for sensitivity and 96.1% for specificity (*p* < 0.01). Variability between studies appeared most influenced by differences in algorithm type (machine learning vs. deep learning), sample size, and geographic region (Asia vs. non-Asia) (Table 3). Publication bias was high, with a *p*-value of 0.000 in Deek’s funnel plot asymmetry test (Figure 5).

Considerable heterogeneity was observed across studies (I^2^ = 94.4% for sensitivity and 96.1% for specificity). Such high heterogeneity is common in diagnostic accuracy meta-analyses due to threshold effects and methodological variation across studies. In the bivariate random-effects model, the overall between-study heterogeneity was moderate (bivariate I^2^ = 68%). The univariate meta regression analysis was performed by algorithm, sample size, and by region (Tables 4, 5).

**Table 4:** Overall subgroup difference tests.

Moderator	Chi-square statistic (χ^2^)	Degrees of freedom (df)	*p*-value
Algorithm type	~1.84	2	0.39
Sample size	6.54	2	0.038
Region	8.56	2	0.014

**Table 5:** Meta-regression using a bivariate random-effects model by algorithm type, sample size, and region.

Moderator	Comparison	Outcome	β (logit scale)	SE	z	*p*	95% CI
Algorithm type	ML vs. DL	Sensitivity	−0.314	0.373	−0.84	0.400	−1.046 – 0.417
		False positive rate	0.544	0.414	1.31	0.189	−0.269 – 1.356
Sample size	≤1,000 vs. > 1,000	Sensitivity	0.705	0.312	2.26	0.024	0.093–1.316
		False positive rate	−0.883	0.337	−2.62	0.009	−1.543 – −0.222
Region	Non-Asia vs. Asia	Sensitivity	−0.891	0.322	−2.76	0.006	−1.523 – −0.259
		False positive rate	0.941	0.341	2.76	0.006	0.273–1.608

Meta-regression was performed using a bivariate random-effects model (Reitsma model). Regression coefficients are reported on the logit scale. DL algorithms, studies with >1,000 participants, and Asian studies served as reference categories.

The bivariate random-effects model showed that algorithm type was not significantly associated with diagnostic accuracy. Compared with deep learning models, machine learning algorithms showed a non-significant reduction in sensitivity (*β* = − 0.314, SE = 0.373, *p* = 0.40) and a non-significant increase in false positive rate (β = 0.544, SE = 0.414, *p = 0.19*). These findings suggest comparable diagnostic performance between ML- and DL-based models. The findings indicated that the study sample size significantly influenced diagnostic accuracy. Compared with studies including more than 1,000 participants, smaller studies (≤1,000 participants) demonstrated significantly higher sensitivity (*β* = 0.705, SE = 0.312, *p = 0.024*) and significantly lower false positive rates (β = −0.883, SE = 0.337, *p = 0.009*). The overall likelihood-ratio test confirmed a significant subgroup difference (χ^2^(2) = 6.54, *p =* 0.038). Meta-regression analysis demonstrated that geographical region significantly influenced diagnostic accuracy. Compared with Asian studies, non-Asian studies showed significantly lower sensitivity (β = −0.891, SE = 0.322*, p = 0.006*) and significantly higher false positive rates (β = 0.941, SE = 0.341, *p = 0.006*). The overall likelihood-ratio test confirmed a significant subgroup difference (χ^2^ (2) = 8.56, *p = 0.014*).

**Figure 6:**
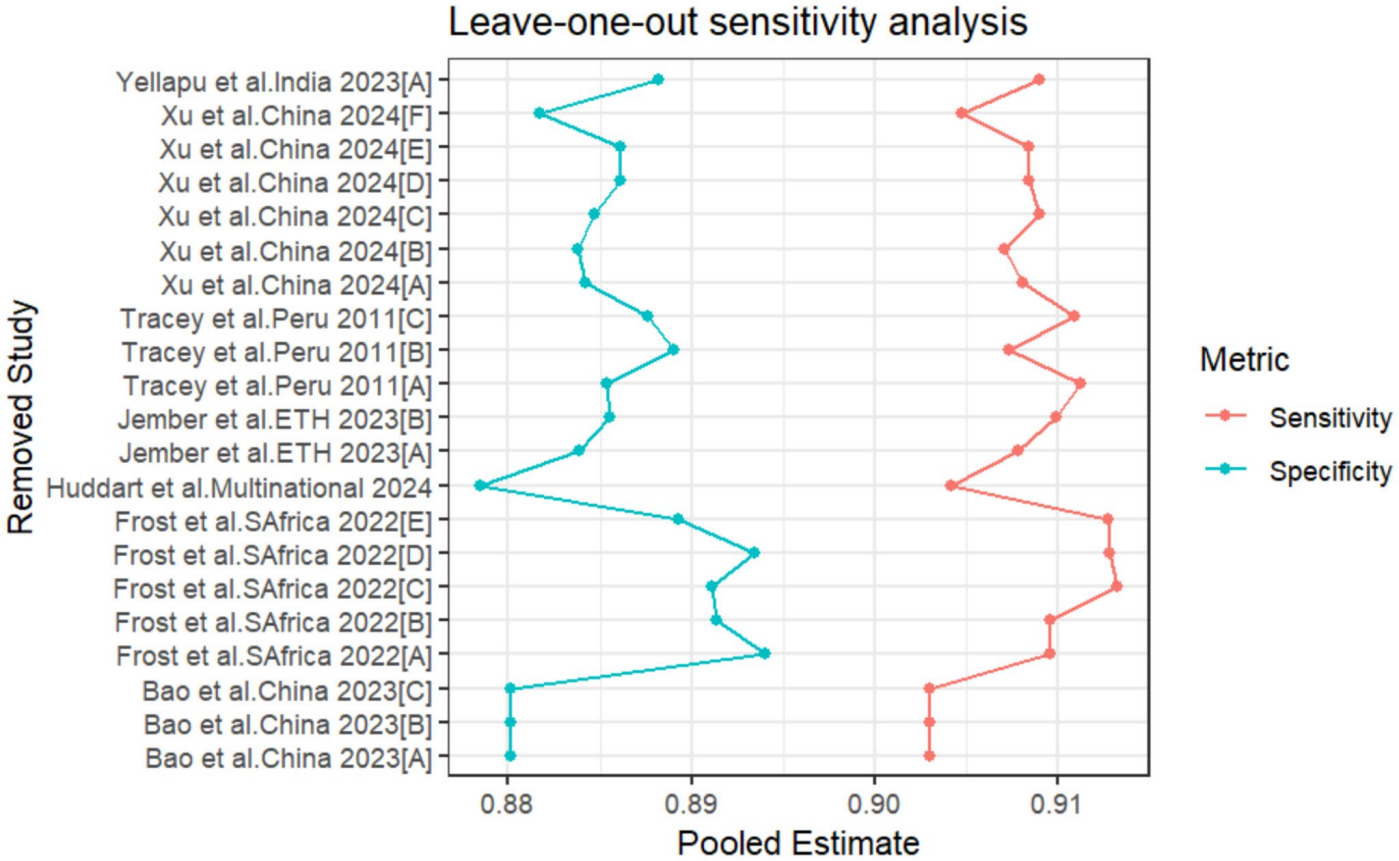
Leave-one-out sensitivity analysis.

Additionally, the leave-one-out sensitivity analysis (Figure 6) showed that exclusion of any individual dataset resulted in only minimal changes in the pooled sensitivity (range: 0.903–0.913) and specificity (range: 0.878–0.894), indicating that no single study had a substantial influence on the overall diagnostic accuracy estimates.

## 4 Discussion

This systematic review and meta-analysis provide a comprehensive evaluation of the diagnostic effectiveness of AI-based models for tuberculosis detection via cough sound analysis. The meta-analysis yielded a diagnostic odds ratio of 81.61 and an area under the curve of 0.9539, with a pooled sensitivity of 91% and specificity of 89%, demonstrating robust overall diagnostic accuracy. Deep learning models demonstrated a pooled sensitivity of 92% and specificity of 91%, while machine learning models showed lower values of 89% for pooled sensitivity and 84% for pooled specificity.

Despite numerous studies on AI-based TB-detection using cough sounds, a comprehensive synthesis of diagnostic accuracy across varied demographics and recording settings is lacking. Existing reviews are restricted in their insights due to varied inclusion criteria and insufficient data reporting. This systematic review and meta-analysis intend to address this gap by preregistering a comprehensive search strategy focused on quantitative diagnostic accuracy, such as sensitivity, specificity, AUC, applying robust appraisal to capture risk of bias and applicability, conducting hierarchical meta-analyses with random-effects models, performing sensitivity analyses and subgroup analyses.

The high diagnostic accuracy reported in this review, with an AUC of 0.9539, with 90% pooled sensitivity and specificity, should be interpreted with caution in the context of real-world applicability in community-based TB detection, particularly in LMICs (World Health Organization, 2023; Yadav et al., 2024; Kapetanidis et al., 2024; Chung et al., 2021; Husssain et al., 2024). The reported performance may reflect favourable results on curated or limited external validation datasets used during the development of the AI algorithm, which possibly raises concerns about generalizability to diverse populations, as recording conditions encountered in routine practice. In LMICs, factors such as variations in age distribution, comorbidity profiles, cough etiology, device quality, and recording environments can substantially alter algorithm performance (Hansun et al., 2025; Mamun, 2022; Alqudaihi et al., 2021; Hosny et al., 2018). If validation datasets lack representative diversity or real-world noise, the high AUC, sensitivity, and specificity observed in development or constrained external tests may not transfer to broader community settings. Moreover, preprocessing choices, feature representations, and the selection of classification thresholds can differentially influence deep learning versus traditional machine learning models, further affecting generalizability across populations and settings.

Additionally, all the DL architectures, including GoogLeNet /ResNet50/Bi-LSTM/ Bi-GRU achieved a high diagnostic accuracy due to their ability to capture complex acoustic patterns directly from either raw or minimally processed data. ML models such as SVM, LR, and SMO achieved a pooled sensitivity and specificity with greater variability in performance, possibly reflecting limitations in manual feature extraction methods (Mamun, 2022; Alqudaihi et al., 2021; Hosny et al., 2018; Rajput et al., 2023; Fang et al., 2021; Oh et al., 2023; Isangula and Haule, 2024; Sahoo et al., 2024). The review indicates that future research should prioritise prospective validation with standardised protocols, including open reporting, in order to determine whether the diagnostic accuracy persists in real-world conditions.

The real-world deployment of an AI-based cough sound analysis approach for TB detection has several potential benefits. It is one of the high-scalability, rapid, community-based, low-marginal-cost screening strategies that can be deployed in doorstep, screen-wide settings, especially in resource-limited regions. This strategy may augment triage workflows to reduce the burden on expensive TB tests and has the ability to leverage large-scale data to improve performance (Dutta et al., 2018; Chauhan et al., 2024; Dubey et al., 2022; Ramadhan et al., 2025). Furthermore, standardisation opportunities across sites through model-based decision support. However, the detection performance is often sensitive to cough aetiology, comorbidity conditions, audio data quality, and recording environment, which are limiting the generalisability of real-world implementation. Therefore, it may require extensive standardisation and robust calibration of the data quality prior to large-scale implementation (Kapetanidis et al., 2024; Dubey et al., 2022; Ramadhan et al., 2025; Jian et al., 2021; Altman and Royston, 2000; Huddart et al., 2024; Jayakumar et al., 2022; Yu et al., 2018). Moreover, in large-scale implementations, the regulatory and ethical aspects concerning the privacy of audio data collection and storage might be important. There is a need for a standard guideline on integrating into existing clinical and programmatic workflows.

The analysis is limited by substantial heterogeneity in study design, recording environments, dataset sizes, and diagnostic confirmation methods. High publication bias was detected (*p* = 0.000, Deek’s Funnel Plot Asymmetry Test), with smaller studies tending to report higher accuracy, suggesting selective publication may have inflated the pooled effect size. Furthermore, the analytical studies could easily overestimate the accuracy. The subgroup analysis was difficult to perform based on the quality assessment of the studies. Additionally, the lack of external validation restricts generalisability in broader clinical settings. Moreover, we restricted inclusion to English-language publications, which may have excluded relevant non-English studies. Grey literature and unpublished datasets were not comprehensively searched, potentially missing additional evidence. Furthermore, although multiple databases were searched, some relevant studies might not have been indexed, and the review relied on reported rather than raw data for analysis.

## 5 Conclusion

AI-based cough sound analysis shows promise as a non-invasive, rapid, and scalable approach for tuberculosis screening, especially in resource-limited settings. However, the current evidence is limited by methodological heterogeneity, risk of bias, publication bias, and insufficient real-world clinical validation. Future research should focus on prospective multicentre studies using standardized recording methods, representative populations, and external validation before routine implementation in TB screening programmes.

## Data Availability

The original contributions presented in the study are included in the article/supplementary material, further inquiries can be directed to the corresponding authors.
